# Medical Lysenkoism

**DOI:** 10.1111/jep.14181

**Published:** 2024-11-06

**Authors:** Steven K. Baker

**Affiliations:** ^1^ Department of Medicine, Divisions of PMR & Neurology Peripheral Neuropathy Clinic, McMaster University Hamilton Ontario Canada

**Keywords:** evidence‐based medicine, ideology, Lysenkoism, medical science, philosophy

## Abstract

Medicine is a compound field composed of science and art. The (necessary) degree to which the latter is involved opens medicine, in particular, to the introduction of ideas which do not, by their very nature, submit to confirmation or confutation as do the various methods of traditional science. This paper explores several ways in which a Lysenkoistic ideology can be interjected into medicine changing the manner in which it interacts with both patients and society. Simply, when ideology supersedes evidence the edifice of Western Medicine comes under direct threat. The exploration of this emerging ideological transition in medicine, and science more broadly, is of critical importance to determine the most salutary path forward.

Medicine has as its chief purpose the minimisation of suffering that attends both psychological and physical disease. Disease—defined presently as that thing which disrupts, impedes, or alters normal anatomy and/or physiology so as to limit function either in the present or, if unchecked, in the future or that condition which compels, by the weight of statistical evidence, prophylaxis. The spectrum of disease ranges from benign, posing no threat to longevity, (e.g., idiopathic alopecia) to lethal (e.g., septic shock). The project of medicine has been to reveal the reductionistic truths of cause and effect within the human body so as to acquire the highest level of pathophysiologic knowledge which can thus best inform potential interventions that might favourably alter the disease‐process or cure it outright. Such an undertaking has, *ab inito*, necessitated a precision of method which is characteristically termed the scientific method. The means by which diagnosis and treatment have largely advanced, notwithstanding serendipity which subscribes neither to method or analysis, is through the rigorous application of the scientific method.^i^ It is here where medicine most reflects science properly understood. However, a physician is more than an amalgam of science and instrumentation. Rather, practitioners of medicine apply their craft to fellow persons thus inviting an element of the arts/humanities into their profession. Medicine is thus a hybrid of science and art. Such an amalgam is particularly susceptible to the incursion of non‐defeasible ideas into what ought to be, broadly construed, reserved for only those facts that emerge from well‐controlled experiments conforming to the rigorous application of the scientific method.

In this context, science and medicine are considered progressive to the extent that they are falsifiable. Indeed, in order for a thing to be falsifiable it must be open to dissent. Sublation and refutation—the very substrate of scientific discourse—must be maintained for if these are lost then the edifice upon which science and medicine sit must necessarily be undermined. Thus, a Socratic agnosticism or epistemic humility^1^ must maintain the central position to facilitate growth in science and medicine. Immured in the concept of humility is a vulnerability to rebuke, correction, and instruction. Medical Lysenkoism^ii^ arises when such facilatatory agnosticism is sacrificed for ideology. The politicisation of medicine, by definition, substitutes the indeterminate goaliii of enquiry‐based knowledge acquisition to the pre‐determined goal of ideology‐based political ends. This may occur by trickle or flood. Lysenkoism then is a form of goal‐oriented negligence to the true objectives of science. If truth‐oriented ethical conduct is not held as the apotheosis of practice then anything which contributes to this infelicitous reordering of priorities derogates truth and besmirches ethics as collateral damage en route to the pre‐defined *telos* of ideology. While the goal of science and medicine, properly conceived, is clear the pathway along which it unfolds is undefined yet trustworthy because, despite the fact that it is unknown, it is constrained and thus guided by methodological rigidity. The hypothesis may be directed by mind and intention but the results, openly interpreted, either refute that which is wrong or affirm that which is right–by slow or saltatory means. Thus, *ex post* progress supports a truth‐dependent praxis whereas *ex ante* progress is truth‐independent praxis. The former must remain normative while the latter rejected as both antinormative and a threat to scientific integrity.

When truth becomes a casualty of Lysenkoistic medicine (and science) the entire project of knowledge acquisition must be abandoned or grotesquely rearranged for the reward of true knowledge is the potential to manipulate, control, and optimise nature for the benefit of humanity. However, the pursuit of knowledge presupposes that there is something to know, something predictable, whether simple or complex, based on nature's order. The existential necessity of knowledge is an ordered world that can be discerned through rational investigation. Such a world‐view mandates that we humbly and incrementally increase our understanding by interacting with reality through the scientific method under the auspices of a rational mind. If the unbiased interpretation of data is vitiated by ideology the ability to understand and control the object of study becomes proportionately impaired. This impairment, rather than causing a noble retreat toward true science may, and appears to be, leading to the ignoble and casuistic doubling‐down on totalising ideology.

In the absence of order there cannot be predictability–the very thing that constant conjuction^2^ requires to empirically understand the sense‐dependent world by inductive inference. If the principle of constancy is violated as Sartre purports though his philosophical adage, *existence precedes essence*,^3^ then the inherent mutability of the latter destabilises the former which makes it unknowable because it then becomes unpredictable. Therefore, existence–that is to say the natural world–must be predictable in order for the scientific project to logically unfold. The retooling of nature through the doctrine of a fictile existentialism requires that one defy reality to define reality. However, the elision of truth leaves in its wake only an illusion of truth. Indeed, the idealist version of the coherence theory of truth does not ontologically differentiate belief from what makes beliefs true.^4^ The only necessary element is that the belief coheres with a reference set of beliefs. This degenerates into an infinite regress because the reference set of beliefs themselves have to be predicated on some other set and so on. However, if the reference set of beliefs do not require a ceaseless seriatim of supporting beliefs then the foundational belief upon which another is based need not be true at all. Its only qualification is that the downstream belief coheres to it. This is in fact a *petitio principi* iv fallacy wherein the truth of a downstream belief is true by virtue of its coherence to an upstream belief which itself determines the truth at issue. The only obvious means by which to stop the regress is to adopt a foundationalist approach to truth. Whereas foundationalism applies to the moral world, physicalism (or naturalism), its loosely conceived material counterpart, applies to the scientific world.^v^ Therefore, physicalism interprets the natural world using that which is quantifiable and minimises the subjective as it is unmeasurable even if it is statistically analysable.^vi^ Such an approach engenders an empirically ordered understanding of the natural world−free of contamination from ideology (i.e., the pure subjectivity of idea). *Au fond*, the physicalist accepts that that there is something objective undergirding their beliefs even if a complete understanding is yet wanting. If the material world is law‐governed then it is open to discovery by scientific enquiry. If the material world is fictile then by its very plasticity it is dispossessed of predictability and cannot submit to or be conquered by rational enquiry. Such indeterminacy must lead to nihilism or totalising ideology for if truth is not objective then it must be fabricated and its fabrication will unfold in a Thrasymachian^5^ fashion at the hands of the most powerful. An appreciation of the origination of this power is not an arcane concept for it can stem from the object (i.e., *discovering* the material world) or it can arise from subject (i.e., *developing* the immaterial world). Those therefore that hold the reigns to this power will drive the enterprise of medicine (and society) toward one or the other.

The power ordinances of Western society have been construed as an oppressive patriarchal competency‐based hierarchy and its actualisation, *inter alia*, through a Protestant work ethicvii is deemed racist yet nowhere are universities, committed as they are to their beliefs, replacing professors, who epitomise hard‐work as exemplified by their PhDs, with students, who are necessarily oppressed because they do not possess these advanced degrees and are ostensibly subjugated by the rigors of deadlines and expectations. Thus, the academy promotes traditional hierarchy yet promulgates, through their faculty mouthpieces, a clarion rallying cry to invert the present cultural power matrix and usher in a new egalitarian anti‐hierarchy. Yet, to the extent that this is achieved it foments the imposter syndrome because one cannot dismantle meritocracy and claim that diversity is its surrogate without introducing some modicum of diffidence. A system that may unwittingly contribute to self‐doubt cannot be the most advantageous to one's well‐being. Indeed, even in ostensibly pure meritocratic settings self‐doubt is writ large in those who are unconvinced of their abilities.^6^, ^7^ Furthermore, the promulgation of a system in which an agenda for a ‘minoritized’ group is promoted seems at best patronising and worst racist. Moreover, minority elevation as an attempt to redress the past requires the ransom payment of ancestral guilt by the present which can only occur if the guilt is said to be suffused through the system as an incurable spectre which though not seen is nonetheless ubiquitous. It is both necessary and sufficient that any system, other than a pure meritocracy, which recognises and gives preferment to ascribed over achieved characteristics, or in any way elevates the ascribed to anything above the token, derogates the merit‐informed decision‐making process. This is a stoichiometric necessity. Thus EDIviii foists an inexorable stylised ‘race‐centrism’ into the algorithm of progress. Unfortunately, in the very name of progress and redress we may be engaging in regress.

Against a system which places 100% emphasis on merit is set any other which incorporates alternative factors that must detract from merit.^ix^ Our current system is not purely meritocratic as evidenced by the advantage conferred by, for example, beauty^8^ and height^9^ which can predict corporate success. If bias is true, it is axiomatic. Its eliminability is at issue and its elimination is as ambitious as it is impossible. This is again a stoichiometric necessity for indeed any measure employed to advantage a given candidate by virtue of a given ascribed attribute must equally, and to an opposite extent, disadvantage another by virtue of that same ascribed attribute. This is an ineluctable fact just as an organic synthesis equation, considering its reactants and products, must balance so too must the decision equation. For every degree to which non‐meritorious factors impinge on the decision matrix, merit‐based weighting must suffer de‐emphasis. If the result is that race, or any of the other discriminator, has been utilised in a manner to disadvantage a candidate, though done with good intentions, it contravenes the Ontario Human Rights Code.^x^

Moreover, the inestimable complexity of intersectionality with its multifarious layers of oppression makes the engineering of equality of outcome or equity simultaneously highbrowed and evitable. Consider the difficulty in ‘proportional representation’^xi^ which if it is to be etymologically consistent must accurately reflect the numerous proportions in a given population. While this may seem straightforward its inherent complexity bears exploration to expose what the project of equity is and what it is not. If intersectionality is to be properly represented, then it must be fully reflective of the variegated heterogeneity of intersection. In a word, it must be exhaustive for if it fails the neglected group will be twice marginalised–once, presumably, by society and once by the mission of diversity. Let us attempt this venerable project therefore with an exacting calculus that should obviate any condemnatory decries from the EDI community. A few simplifying assumptions are required.xii Let all areas of marginalisation be expressed as a tripartite (i.e., high, medium, low; strong, average, weak; severe, moderate, mild) with the exceptions of sex which will be dichotomous, race which will be quadripartite (i.e., white/Caucasian, Mongoloid/Asian, Negroid/Black, and Australoid), religion which will be component 13,xiii and gender identity component 16.xiv

Proportional Intersectional Diversity (PID)^xv^:

1. Attractiveness x 3 (i.e., high–medium–low)
2. Weight x 3 (i.e., high–medium–low)
3. Sex x 2 (i.e., male–female)
4. Gender identity x 16
5. Religion x 13
6. Race x 4
7. Intelligence x 3 (i.e., high–medium–low)
8. Height x 3 (i.e., high–medium–low)
9. Affability x 3 (i.e., high–medium–low)
10. Sociability x 3 (i.e., high–medium–low)
11. Communication x 3 (i.e., strong–average–weak)
12. Curriculum Vitae x 3 (i.e., strong–average–weak)
13. Disability Status x 3 (i.e., severe–moderate–mild)

PID = 2 × 3^9^ x 4 × 13 x 16 = 32,752,512.

The problem is worse. Race has been overly simplified for the purpose of this exercise. The World Health Organisation prefers to address variations in peoples as per ethnicity of which there is estimated to be 5000. Notwithstanding the WHO's extreme estimate, if a more moderate estimate of ethnic groups is employed (i.e., 518)xvi and is substituted for race (i.e., 4) the PID estimate would explode to 4.2 × 10^9^. As the world population is only 7.9 × 10^9^ it would appear that a comprehensive assessment of PID would essentially approximate the individual, which indeed a rational evaluation would logically lead one to conclude from the outset. It must, as a result of the above, be concluded that the challenge to human resources and indeed the general project of hiring, to effectuate equity, mathematically understood, is insuperable.

Notwithstanding the foregoing logical hurdle another obvious dilemma for equity would seem to be the ‘singular position’. However, this appears to be readily resolved through the de‐intersectionalization of all minorities into an isomorphic group identified not by what they *are* but by what they *are not* (i.e., nonwhite). Thus, the numerical problem of limited postings/positions forces a forfeiture of the equity mission via an oxymoronic tacit amalgamation of minoritized identity groups under the indecorous epithet of ‘nonwhite’ such that the meaning of their inclusion into the group from which the candidate will be selected is still informed by the prevailing white majority. A more devaluing calculus could not be conceived for the value of an individual from a minority group is found from within and not predicated on a racial comparison without. The value of a minority group or individual ought to have no contingency or reference to the Caucasian race. But the collectivist sees not the one only the many.

A related challenge must be honestly confronted when attempting to engineer equity throughout the ostensible institutionalised matrices of oppression. It is the vexing question of end‐point. How do the summated actions of multiple actors across society (academe, science, and medicine in particular) determine when equity has been achieved? This question plagues all forms of activism. The more deliberate the activism promotes an imagined outcome the more adamantine it must become to secure its final results which are hardest to realise by dint of their extremity. Therefore, this sets up a great paradox, wherein after so much may have been accomplished toward the achievement of the initial, admittedly noble, goals the final territory to be conquered appears less amenable to persuasion and more demanding of fiat. A movement, it would seem, cannot certainly rest on any guarantee of results, towards the end of its mission, that might now depend on an enlightened and novel or modified social contract. Rather, having no trust in the goodwill of the perpetrators who are assigned historical culpability for the problem in the first place, the cry of outrage and the accusations of abuse and oppression grow louder the closer the ill‐defined finish line is approached.xvii The power of propinquity then is not to assuage the activism but to in fact enrage it so that yet another paradox is created—the greater the acquiescence the greater the accusation—sending the general populous into ever deeper confusion. This then establishes a dishonest Sisyphean praxis of stoking culpability with every act of capitulation.xviii As the activist objectives are steadily accomplished the indictments against society intensify. It would appear that the perpetuity of a given movement's terminal crescendo phenomenon has at least as much to do with a self‐preservatory impulse as it does for the actual cause itself. In this conception, the politicisation of a socially directed objective, through the praxis of activism, wittingly or unwittingly, fosters the tendency to maintain social power independent of, and in fact despite, its proximity to its stated goals. This creates another type of social movement regress characterised by the receding goal‐post phenomenon. If the objective end‐point is ill‐defined, which it admittedly is,^10^ ^,^ xix then the idea that the goals are near‐at‐hand foments great action despite the inability to close down on the goal because it is neither fixed in space or time. There is, thus, a futile storm pressing toward an elusive goal mired in the miasma of ill‐description and recession.

Any infiltration of science or medicine by such doxy would devastate their unadulterated objectives. In particular, the practice of medicine as the privileged, n of 2, relationship between doctor and patient would become subservient to a collectivised project to eliminate disparate outcomes under the guise of equity. Equality of outcome is a pernicious governing principle for the sanctuary of the clinic must be preserved to permit the doctor to best manage her patient, respecting Hippocratic and deontological principles. Critical theory is an interpretive lens initially conceived to abolish all that is. Yet this Hegelian‐Marxian‐Marcusian fatalistic infatuation with collapsing the bourgeoise West must include the destruction of the scientific project which is substantially indebted to the White European diaspora of the Enlightenment who inhabited the halls of venerated faculties of old and ‘infelicitously’ stole the accolades for their discoveries. If the edifice of medicine is to be castigated as a product of invidious colonialism, in what manner should it be dismantled and by whose hubris are such decisions to be made? And to what ill‐effects?

The framing of medicine that conveys a duty to the collective, while this can be broadly understood as one of the purposes of Public Health, through a medicalized deindividuation, is inimical to the practice of medicine in the traditional care model. However, advancing genetic knowledge is increasingly realising the potential clinical utility of genome‐based personalised medicine. Thus, the direction of scientific progress (i.e., individual medicine) is running counter to political trends (i.e., collective medicine). It should be generally uncontested, amongst practising physicians, that politics and medicine should be deemed irrevocably immiscible because when they are politics prevails. This is acutely dangerous particularly in the age of genetics where genetic algorithms may be deployed in service to equality of outcome leaving the practice of individual medicine irreparably maimed and placing the lives of the groups needing to be equalised at risk.^xx^ Respecting group outcomes at the expense of the individual, while it may achieve collective ideological goals, maligns all ethical principles including autonomy and freedom of conscience. As Sharrer^11^ rightly states, ‘[p]ersonalized medicine, with its enhanced capacity to access the individuality of illness, must have a continuously evolving feedback mechanism–the most important element being the physician–patient relationship–which is its ethical footing’. To lose the centrality of this footing, ethical practice cannot be carried out and the physician devolves, one mandate at a time, into an apparatchik of the ideological promulgators. This is in fact, one of the main dangers of socialised medicine—the well inculcated idea that government does and ought to control medicine—such that when corruption arrives, its recognition by a lulled populous will be, like the wolf licking the frozen blood from an inverted knife in the ice, too late. Slowly, as with the precision and patience of medieval cathedral masons, the government issues various healthcare regulations, which individually are neither onerous nor imposing, but the incessant creep of the same paralyses one's will to resist and stupefies one's insight as to the time at which its motives would appear to transition from fiduciary to fiscal to the fiat of soft then finally hard tyranny. The inertia of such acceptance encourages the arrogation of power as has recently been exercised through the sequential actions of the state's enactment of emergency measures and Public Health's transfixion on antiviral sanitation and ‘stopping the spread’. The SARS CoV2 pandemic has well proven this thesis.^12^ The precautionary principle, in the main then if left to government's discernment, will always sacrifice the individual for the collective with the rhetoric that safety should always supersede freedom. Thus, with the promise of a fortnight's lockdown did the lure of freedom henceforth guarantee the loss of that very freedom.

A cautionary lesson from less than a century ago should cause a circumspect pause with regard to the medicine‐government hybrid, under whose direction, a progressive enlightened social engineering programme of eugenics was unleashed.xxi From utter commitment to utter catastrophe the relentless materialisation of man, conceived of as an object of study not a subject of knowledge, fosters a contempt for those who align not with the prevailing ideology. Brother's 1950 assessment of the emerging scientocracy seems eerily apropos to the present and bears repeating.^13^

*It has been stated that there is no sign that any progress in mental capacity has taken place since the days of the ancient Greeks; and further, it has been asserted that the human race is succeeding in producing proportionately fewer persons of eminence than did these same ancient Greeks: briefly, what is meant is that the human race is actually morally and intellectually worse off now than it was 2000 years ago*.

Malcolm Muggeridge captured this sentiment of a misguided academy even more poignantly.^14^

*So, the final conclusion would surely be that whereas other civilisations have been brought down by attacks of barbarians from without, ours had the unique distinction of training its own destroyers at its own educational institutions, and then providing them with facilities for propagating their destructive ideology far and wide, all at the public expense. Thus, did Western Man decide to abolish himself, creating his own boredom out of his own affluence, his own vulnerability out of his own strength, his own impotence out of his own erotomania, himself blowing the trumpet that brought the walls of his own city tumbling down, and having convinced himself that he was too numerous, laboured with pill and scalpel and syringe to make himself fewer. Until at last, having educated himself into imbecility, and polluted and drugged himself into stupefaction, he keeled over–a weary, battered old brontosaurus–and became extinct.*

It is of no surprise that atrocities of reasoned calculation should arise and history bears witness to the same; the challenge is to resist the inertia that begins to coax society in directions that it ought not go. Often, as was certainly the case with eugenics and sterilisation, the afflatus of condemnatory insight came too late only when its failures were writ large and society was not measurably benefitted. Thus, the task to retain ethically rigorous side‐constraints immuring the project of medicine must remain an utmost focus second only to that of healing for when the healers turn harmers the greatest harm is done. In this light then, so long as the mission of healing continues to inspire purpose medicine need not safeguard against science itself but against the misapplication and perturbation of science by unfettered politicisation (i.e., Lysenkoism).

Against gene‐realism, which gave birth to the genetic revolution^15^ and continues to inform and transform every branch of medicine, there is an inescapable contradiction in the dissolution of man and woman as genetic categories.^16^ The same value‐independent gene‐realism extoled for the counsel it provides the parents of a child with cystic fibrosis^17^ is excoriated as phobic and bigoted when attention is turned to another area of the genome. Therefore, while science‐driven gene‐realism has radically improved the pathophysiologic understanding of disease and thus the practice of medicine it has simultaneously been renounced through the legerdemain of value‐dependent antirealism as being unconnected to phenotype. It is only in this idea‐driven deconstructivist reality where the maternal inheritance of mitochondria and the paternal inheritance of the Y‐chromosome (and the SRY gene) though crucial to hapolotype mapping and genetics in general can be disconnected from their Watsonian and Crickian materialism and thus etiolated by hypostatisation into a vapid idea of progressive gender ideology. In the attempt to construct a new reality, devoid of all that sympathises with equipoise and harmony, do they, the vanguards of change, create the very soil from which nothing of substance will grow because if it has no base neither can it have nutriments.

Ideological capture obfuscates the mission of medicine which must unassailably and intransigently defend the promise to do no harm while fulfilling its great commission to solve disease. Understanding medicine as a synthesis between the sciences and humanitiesxxii contextualises the difficulty in warding off idea from fact. The transgender movement (TGM) exemplifies this awkward chimera of fact and idea. Gender fluidity, *inter alia*, is marketed as a guarantee of sexual liberation and self‐actualisation. As with many things claims are made true by the consequences of their actions. Consider, the five major life stressors^18^ which hold in common the adaptation to major change. Thus, by syllogistic certainty the exposure, and in particular the repeated exposure, to important life‐altering change is stressful.xxiii If the situation of a geographical move is stress‐inducing could it not be logically inferred that being in a perpetual vacillatory state regarding what one's essence/gender might be on any given day might also be a source of immense stress? A move to another address is stressful due to multiple externalities but a move between identities would appear to be fundamentally destabilising for one's internalities allow one to know oneself. However, if these constituent essences are capricious the Delphic admonition ‘to know thyself’ would be of a difficulty not originally conceived of by the oracle. Such Heraclitian gender flux would seem to serve as a source of instability rather than stability. This in turn may, though calculably inevitable, cause or worsen anxiety and depression. If this be true, even in a singular instance, the mission of medicine has failed under a Lysenkoistic lodestar for rather than thoroughly studying each patient's dilemma with a rogatory truth‐centric scepticism there is an impetuous ideological decision to assent to the neo‐identity.

The Mayo Clinic in its supportive model cannot see through its patent activist approach as it states, ‘Behavioural therapy isn't intended to alter your gender identity. Instead, therapy can help you explore gender concerns and find ways to lessen gender dysphoria’.^19^ Furthermore, the American Psychological Association's 2015 practice guideline^20^ states, ‘[w]ithin the last two decades, there has been a significant increase in research about TGNCxxiv people. This increase in knowledge, *informed by the TGNC community*,xxv has resulted in the development of *progressively more trans‐affirmative practice* xxvi across the multiple health disciplines involved in the care of TGNC people’.^22^, ^23^ However, this unidirectional affirmation, especially of the kind that would lead to reassignment surgery with the irrevocability of metoidioplasty and vagino‐vulvoplasty, ignores the problem of measurement, dismisses epistemic uncertainty, and conflates certitude for infallibility. Indeed, Wittgenstein cautioned against certainty when the statement, ‘I am certain’ becomes, ‘I cannot be wrong’.^24^ If epistemic humility implies a vulnerability to error and counter‐argument then epistemic arrogance, through the very process of refuting the same, invalidates itself by (1) the impossibility of gnosticism or (2) the refusal to accept the statistical ineluctability of type I and type II errors. Thus, as a statistical necessity, an affirmatory model must be subject to error. In so far as this is true an affirmatory model cannot be absolute because it would contravene the law of noncontradiction. The physician's mandate is to service the patient and not the direct the patient into service of an activist ideology. The pollution of non‐Hippocratic ideology into the medical enterprise proportionately erodes the safety of the patient and the sanguinity of the public. To the extent this occurs the Kantian categorical imperative, to treat humans as ends and not means, will be breached.^25^, ^26^ Activism thus parochially serves to advance its cause independent of the patient's best interest as that interest has been subsumed by the activism and a priori determined to be the *summum bonum*. Therefore, ‘woke’ medical Lysenkoism simultaneously eschews Kant and embarrasses statisticians.xxvii

Similarly, the application of logic is central to the task of diagnosis. If logic is abrogated diagnosis is impossible. Consider the syllogistic breakdown between ideology and practice demanded by the TGM.

Ideology:

Premise A—Gender is purely a social construct.

Premise B—Gender has no connection to biologic sex.

Conclusion—Secondary sexual characteristics are immaterial to gender.

Practice:

Premise A—Gender is purely a social construct.

Premise B—Gender has no connection to biologic sex.

Conclusion—Sex reassignment surgery is necessary to align with a new gender identity.

The ultimate rejection of Nature is to deny the categories of man and woman altogether. For the purposes of ideological consistency, the penultimate rejection of Nature is to affirm sexual indeterminacy by retaining the original hardware as proof of its immateriality to the newly assumed gender identity. Indeed, many do reach this conclusion. For those who haven't the same argument applies to endocrine manipulation. One cannot claim that sex hormones are meaningless and then attribute meaning to them by doubly endorsing their biologic importance–first by blocking the actions of the endogenous hormones and second by prescribing exogenous cross‐sex hormones. Thus, the combined actions of cosmetic surgeons and endocrinologists expose their lack of philosophical perspicuity regarding the law of noncontradiction. By reversing the order of the no harm–help couplet into help–no harm they may be causing harm in the attempt to help. But to the extent that this cannot honestly be studied due to ideological capture or threat of cancellation the truth cannot be known. Thus, for the cause of medicine it cannot maintain its mandate of *primum non nocere* as it cannot know if it cannot study.

A final consideration of utmost importance in the practice of medicine is the differentiation of what is necessary to a disease and what is accidental to it. As Sydenham eloquently stated, ‘He who describes a disease must take care to distinguish the symptoms that necessarily accompany it, and which are proper to it, from those that are only accidental and fortuitous, such as those that depend on the temperament and age of the patient’. This naturalist/physicalist approach therefore, in a certain sense, relegates the patient as an external fact—the attempt to separate the reaction of the mind from the body's illness. A patient's voluntary reactions to a disease are neither necessary nor sufficient to provide the relevant diagnostic clues. By contrast, the involuntary reactions of the splanchnia, pleura, and/or cardia are both necessary, and, in the hands of the perspicacious diagnostician, sufficient. However, elements of the patient which are neither necessary nor sufficient are accidental and though accidentals may have some bearing on diagnosis they do not form the sole basis of it. In this regard, race essentialism as propounded in critical race theory and its offshoots of EDI seek to rank, much higher than it ought to be, the status of race. Race essentialism expressed through the project of equity therefore elevates the accidental into the diagnostic and/or management algorithms where it should be considered only to the extent that known race‐specific genetic tendencies should raise or lower caution about biologic (i.e., disease and pharmacodynamic) idiosyncracies.

While medical Lysenkoism does not preclude the practice of medicine it does require that the bastion of evidence‐based medicine (EBM) be replaced by ideology‐based medicine (IBM). Whereas science generally, and EBM specifically, hold truth to be discoverable based on the preponderance of randomised‐controlled trial evidence, IBM is exercised in service to pre‐determined ends. Thus, the former are falsifiable by virtue of their epistemic humility^1^ acknowledging the measurement problem adduced by Djulbegovic.^27^ By contrast, IBM exorcises humility in a gnostic takeover of an intellectual pursuit immolating truth and promoting confirmation bias. Indeed, confirmation bias has now been formally reified into the concept of reflexivity–a purely subjective process whereby a researcher ‘can account for the significance of the intertwined personal, interpersonal, methodological, and contextual factors that bring research into being’.^28^ This is fundamentally misguided as research is not ‘ontogenic’ but rather ‘epistemogenic’. Indeed, the subjectivist‐inductivist paradigm lauds the subjective in blind deference to an analytical method which is haplessly indifferent to the problem of induction. In their own words, ‘the ideal of “unbiased”…algorithms inevitably hearkens to objectivism and may neither be achievable nor desired from a subjectivist perspective’.^29^ The militation against such an invidious praxis must be central to the project of medicine. Competing theories which do not place objective pathophysiology as the cynosure of investigation err in the inflation of the accidentals and the elision of the necessaries. Thus, the incorporation of the humanities into science‐laden medicine must be constantly on guard against both the incursions of ideology and the allure of ultracrepidarian excursions. The collision of the incursive and excursive into medicine under the auspices of the state will produce an authoritative iatrocracy. Logic and reason under the governance of deduction and induction expressed through the scientific method must inform the abductive endeavour of medicine whose sole pursuit is individual health.xxviii Any praxis that does not conform to these principles must be rejected.

The intrinsic value of humans is based not on tribal/group identity but on an ineffable truth that all people have an unearned prepossessing value that cannot be inflated or deflated (by race or any other achieved or ascribed attribute). The critical race theory (CRT) premise of ubiquitous racism exemplified by the statement, ‘It is not a question of whether racism happened but rather how it manifested’, is actually a complex question fallacy. It is the gnostic assignment of guilt via the assumption of a truth in the absence of evidence. The classic example given in logic courses is, ‘When did you stop beating your wife?’ The question makes an assumption that the interlocutor does beat his wife and this may be, and hopefully is, untrue. If untrue then the question is utterly invalid. Thus, whites being accused of implicit racism, as CRT purports, is no more logically valid than accusing all men of being spousal assaulters. In fact, this is referred to as a fallacy of composition which attributes to the whole that which is applicable only to a part (i.e., fallacy of synecdoche). Thus, the organising principles of CRT are guilty of several fallacies. Any theory which espouses unmitigated fallacies cannot inform or participate in the scientific method, as an interpretive lens, for it will ruin rather than rarefy the project of medicine which needs to establish its gaze upon the fixed objectively discoverable natural world not the fictile and fallacious.

## CONFLICT OF INTEREST STATEMENT

The author declares no conflicts of interest.

## Data Availability

Data sharing not applicable to this article as no datasets were generated or analysed during the current study.

## Footnotes

i Thomas Kuhn discusses, at length, in his book *The Structure of Scientific Revolutions*, the power of paradigms to fundamentally shape both the formulation of premises and the subsequent interpretation of data. Max Plank originated the notion that progress depends not on the weight of evidence and argumentation but rather on the deaths (or retirement) of those who developed and promulgated the prevailing paradigm. This has been popularised in the phrase, ‘Science progresses one funeral at a time’. The gravamen of this phenomenon of fatality‐dependent progress is not that the minds of those already made‐up are impossible to change but that the purity of the scientific method is fallible because it is at once the victim of incomplete knowledge and ideological contamination. It is the contaminability that serves as the basis of this paper.

ii Henry Rosovsky, a Harvard economist, in his 1990 book, *The University: An Owner's Manual*, cautioned his readers to, ‘[n]ever underestimate the difficulty of changing false beliefs by facts’ (The University: An Owner's Manual, Prometheus 2008, p. 149).

iii The scientific result is not determined a priori but rather develops by *a posteriori* analysis as data are reconciled with a paradigm to either confirm, modify, or refute it.

iv Begging the question fallacy wherein the premise informs the conclusion which produces a nonanswer and thus the question remains at large.

v My point here is simply to illustrate that without a definite, concrete, or objective basis for understanding a thing, be it moral or material, it is elusive and subject to whimsical change making it difficult if not impossible to know. Right being suddenly wrong is equally destabilising for a system of thought as a cell membrane to be declared hydrophobic in one moment and hydrophilic in the next. There must, in other words, be constancy and predictability. If knowledge of the operation of the natural world is fictile then it is necessarily futile. C.S. Lewis addresses this very point in *Abolition of Man*, Ch. 1 ‐ Men without Chests; p.6.

vi The application of statistical analysis, though mathematizing the subjective, does not make it real or empiric.

vii See https://www.miamiherald.com/news/nation-world/national/article244309587.html.

viii Equity, diversity, and inclusivity.

ix MBS = 1.0 _m+_; CS1 = 0.9 _m+_ + 0.1_r_ = 1.0_m–_; CS2 = 0.8_m+_ + 0.1_r_ + 0.1 _s_ = 1.0_m–_; CS3 = 0.6_m+_ + 0.2_r_ + 0.1_gi_ + 0.1 _fs_ = 1.0_m–_; MBS, merit‐based system; CS1‐3, competing system 1‐3; m +, pure merit; m–, not pure merit; r, race; s, sex; gi, gender identity; fs, fat status.

x Employment: 5 (1) Every person has a right to equal treatment with respect to employment without discrimination because of race, ancestry, place of origin, colour, ethnic origin, citizenship, creed, sex, sexual orientation, gender identity, gender expression, age, record of offences, marital status, family status or disability. R.S.O. 1990, c. H.19, s. 5 (1); 1999, c. 6, s. 28 (5); 2001, c. 32, s. 27 (1); 2005, c. 5, s. 32 (5); 2012, c. 7, s. 4 (1). (https://www.ontario.ca/laws/statute/90h19)

xi Diversity is a state or condition that reflects the broad ‘mix’ and layers of differences in any community. In the university setting, compositional diversity refers to the numeric and *proportional representation* of different peoples, across many intersecting dimensions of sociocultural group identities. Source: McMaster Faculty of Health Sciences Diversity and Climate Survey 2021.

xii This immediately relegates the equation to failure because all assumptions are faulty (as even Einstein established with his cosmological constant (Λ) in his field equations of general relativity to account for a non‐expanding universe).

xiii (1) Christianity, (2) Judaism, (3) Hinduism, (4) Buddhism, (5) Islam, (6) Jainism, (7) Baha'i, (8) Sikhism, (9) Zoroastrianism, (10) Confucianism, (11) Shintoism, (12) Taoism, and (13) Scientology.

xiv
https://www.medicalnewstoday.com/articles/types-of-gender-identity#gender-as-a-spectrum

xv All areas of potential bias and discrimination.

xvi
https://en.wikipedia.org/wiki/List_of_contemporary_ethnic_groups

xvii Such a phenomenon has some parallel to exercise training as early fitness is relatively otiose but later higher level fitness becomes bitterly operose.

**xviii:** This zeitgeist is also borne out in ‘cancel culture’ wherein an apology often secures cancellation as admission of guilt justifies, according to this worldview, a definitive redress which becomes necessary as the axiology of forgiveness has been bankrupted in a post‐truth society.

xix Adorno, *Negative Dialektik*, pp. 206‐7. In this work Adorno, a prominent Frankfurt School Critical Theorist, takes the view that utopia can only be negatively conceived and not positively envisioned. In other words, through a step‐by‐step dialectical dismantling of Western culture the utopia, though lacking any positive vision, will alchemically emerge just as protochemists believed the alembic would transform base metals into gold. Thus, only a scientific materialist's economic materialism would envision a reality‐defying process capable of such transformation, but this is exactly what the utopianists of present are so deeply committed to. The Gramscian maxim of, “pessimism of the intellect, optimism of the will” should more rightly be, “obfuscate the intellect, nonplus the will.”

xx This applies to groups above and below the nebulous mean to which they are to collapse toward.

xxi The entire movement was predicated on the duty of the unwanted one to sacrifice themselves for the good of the whole. This is, in essence, the communistic framework.

xxii Science understood as the world of falsifiable (emerging) fact and humanities understood as the world of non‐falsifiable ideas.

**xxiii:** A: Major life change is stressful; B: X is a major life change; C: X is stressful.

xxiv TGNC, Transgender and gender nonconforming.

xxv But this community is most likely to promote activist agendas subject to confirmation bias which has been well documented, in a like manner, with the myalgic encephalomyelitis community.^21^

xxvi This makes the implicit assumption that trans‐affirmation is the best medical practice without a reference to evidence from scientific literature.

**xxvii:** Adapted from, Baker SK. Activism contravenes consent. J Eval Clin Pract. 2023 Sep;29(6):880‐882. doi: 10.1111/jep.13880. Epub 2023 Jun 4. PMID: 37271977.

**xxviii:** This is certainly not to suggest that data from the collective (i.e., EBM) should not inform the practice of individual medicine. Modern medicine, properly understood, uses the collective in service to the individual not the individual in service to the collective.
